# Encapsulation of Recombinant MOMP in Extended-Releasing PLGA 85:15 Nanoparticles Confer Protective Immunity Against a *Chlamydia muridarum* Genital Challenge and Re-Challenge

**DOI:** 10.3389/fimmu.2021.660932

**Published:** 2021-04-14

**Authors:** Rajnish Sahu, Saurabh Dixit, Richa Verma, Skyla A. Duncan, Lula Smith, Guillermo H. Giambartolomei, Shree R. Singh, Vida A. Dennis

**Affiliations:** ^1^ Center for NanoBiotechnology Research, Department of Biological Sciences, Alabama State University, Montgomery, AL, United States; ^2^ Instituto de Inmunología, Genética y Metabolismo (INIGEM), Consejo Nacional de Investigaciones Científicas y Técnicas (CONICET), Universidad de Buenos Aires, Buenos Aires, Argentina

**Keywords:** *Chlamydia muridarum*, major outer membrane protein, nanovaccine, PLGA nanoparticles, neutralizing antibodies

## Abstract

Recently we reported the immune-potentiating capacity of a *Chlamydia* nanovaccine (PLGA-rMOMP) comprising rMOMP (recombinant major outer membrane protein) encapsulated in extended-releasing PLGA [poly (D, L-lactide-co-glycolide) (85:15)] nanoparticles. Here we hypothesized that PLGA-rMOMP would bolster immune-effector mechanisms to confer protective efficacy in mice against a *Chlamydia muridarum* genital challenge and re-challenge. Female BALB/c mice received three immunizations, either subcutaneously (SC) or intranasally (IN), before receiving an intravaginal challenge with *C. muridarum* on day 49 and a re-challenge on day 170. Both the SC and IN immunization routes protected mice against genital challenge with enhanced protection after a re-challenge, especially in the SC mice. The nanovaccine induced robust antigen-specific Th1 (IFN-γ, IL-2) and IL-17 cytokines plus CD4^+^ proliferating T-cells and memory (CD44^high^ CD62L^high^) and effector (CD44^high^ CD62L^low^) phenotypes in immunized mice. Parallel induction of antigen-specific systemic and mucosal Th1 (IgG2a, IgG2b), Th2 (IgG1), and IgA antibodies were also noted. Importantly, immunized mice produced highly functional Th1 avidity and serum antibodies that neutralized *C. muridarum* infectivity of McCoy fibroblasts *in-vitro* that correlated with their respective protection levels. The SC, rather than the IN immunization route, triggered higher cellular and humoral immune effectors that improved mice protection against genital *C. muridarum.* We report for the first time that the extended-releasing PLGA 85:15 encapsulated rMOMP nanovaccine confers protective immunity in mice against genital *Chlamydia* and advances the potential towards acquiring a nano-based *Chlamydia* vaccine.

## Introduction

*Chlamydia trachomatis* is an intracellular pathogen and recognizably the most common agent of bacterial sexually transmitted diseases globally, with over 100 million new cases per year (1, 2). *Chlamydia* causes a wide range of damaging reproductive, clinical, and immunological sequelae in humans, especially in females, but there are still no commercial vaccines for clinical applications. There is a broad consensus that developing a highly effective *Chlamydia* vaccine to eradicate the disease and reduce global infection cases is urgently warranted. Both attenuated or inactivated whole organisms’ vaccines supposedly can induce adequate protection against many pathogens since they contain an array of antigens (3). Unfortunately, some pathogens exercise immune evasiveness and may require different approaches to deliver their antigens (3) to induce adequate protection. Nowadays, purified or recombinant antigens as subunit vaccines are becoming more desirable, mainly due to their safety (1, 4). For many years, the major outer membrane protein (MOMP) of *Chlamydia* have been promising and remains the highly accepted antigenic vaccine target (2, 5). However, one reason for the lag in developing a *Chlamydia* subunit vaccine, such as MOMP, is perhaps, the lack of a robust adjuvant-delivery system that can provide a prolonged targeted-delivery and bolstering of protective immune responses (6, 7).

Failure to develop vaccine constructs against various human infectious pathogens has resulted in exploring nanoparticles as alternative delivery platforms. Biodegradable polymeric nanoparticles are being explored intensively for delivering biomolecules (8) such as drugs and vaccines due to their protective, self-adjuvanting, slow-releasing, and flexibility in the delivery routes (6, 7, 9–11). Previously, we showed that PLA-PEG [poly(lactic acid)-poly(ethylene glycol)]-encapsulating M278 (MOMP peptide) triggered robust *Chlamydia*-specific immune responses and afforded protection against a homologous genital challenge in immunized mice (10). We also reported that recombinant MOMP (rMOMP) encapsulated in PLGA [poly (D, L-lactide-co-glycolide)] 50:50 nanoparticles potentiated *Chlamydia*-specific adaptive immune responses in immunized mice (12). Recently, we aimed to enhance the immunogenic potential of rMOMP by its encapsulation in the biodegradable extended-releasing PLGA 85:15 nanoparticles (PLGA-rMOMP) to serve as an adjuvant-delivery platform. Evaluation of the new PLGA-rMOMP nanovaccine revealed that it augmented endosomal processing, activated and boosted dendritic cells (DCs) responses, including Th1 cytokines and MHC class II molecules. We also observed the bio-distribution of PLGA-rMOMP to lymph nodes of mice by *in vivo* live imaging and enhancement of *ex vivo* T-cells and total IgG antibody immune responses, which were higher by the subcutaneous (SC) than intranasal (IN) immunization route (13).

Based on the above findings, we hypothesized that PLGA-rMOMP would bolster immune-effector mechanisms to confer protective immunity against a *Chlamydia muridarum* genital challenge and re-challenge. Testing of our hypothesis was conducted in female mice using the SC and IN immunization routes. Our results revealed that SC and IN immunization with PLGA-rMOMP conferred protective immunity against genital *Chlamydia* by bolstering systemic and mucosal antigen*-*specific cellular and humoral immune effector mechanisms. Here, we discuss our results, emphasizing the extended-releasing PLGA 85:15 adjuvant-delivery platform for a *Chlamydia* MOMP nano-based vaccine.

## Materials and Methods

### Reagents

*C. muridarum* [strain Nigg II; previously called *C. trachomatis* mouse pneumonitis (MoPn) biovar] expressed as inclusion forming units (IFU/mL) was purchased from Virusys Corporation (Taneytown, MD). The mouse-derived McCoy fibroblasts cell line and Dulbecco’s Modified Eagle’s Medium (DMEM) with high glucose and L-Glutamine were both purchased from American Type Culture Collection (ATCC) (Manassas, VA). PLGA polymer (85:15 poly-lactide: poly-glycolide), dichloromethane (DCM), polyvinyl alcohol (PVA), and mitomycin-C were purchased from Sigma-Aldrich (St Louis, MO). ELISA MAX™ Deluxe kit for interferon-gamma (IFN-γ), interleukin-2 (IL-2), and interleukin-17 (IL-17) were purchased from BioLegend^®^ Inc. (San Diego, CA). RPMI-1640 with GlutaMax™ and HEPES, heat-inactivated fetal bovine serum (FBS), ACK lysing solution, and antibiotic-antimycotic were all purchased from Life Technologies (Grand Island, NY). Anti-CD 90.2 magnetic beads and MACS columns were purchased from Miltenyi Biotech (Auburn, CA). CellTrace™ CFSE (carboxyfluorescein succinimidyl ester) cell proliferation assay kit (C34554) and Remel™ PathoDx™ *Chlamydia* culture confirmation kit (R62210) were purchased from Thermo Fisher Scientific (Waltham, MA). The Fc block anti-CD16/32 antibody (BD:553141), fluorochrome-conjugated antibodies: CD3-APC-Cy7 (BD:560590), CD4-PerCP-Cy5.5 (BD:550954), CD62L-APC (BD:553152), CD44-PE (BD:553134), and Opti-EIA sets for interleukin-10 (IL-10) were obtained from BD-Biosciences (San Jose, CA). Medroxyprogesterone acetate (Depo-Provera) was purchased from Pfizer (New York, NY), and cycloheximide was obtained from EMD Biosciences (La Jolla, CA).

### Nanovaccine Formulation

The rMOMP (14) was encapsulated in extended-releasing PLGA 85:15 biodegradable nanoparticles to obtain the PLGA-rMOMP nanovaccine as reported (13). Briefly, PLGA 85:15 (300 mg) was emulsified in DCM, followed by the addition of 2 mg of rMOMP, homogenization, and then the addition of 1% PVA. The resulting double-emulsion was gently stirred overnight at room temperature (RT) to evaporate the organic solvent, harvested by ultracentrifugation, washed, and then lyophilized in the presence of a 5% trehalose solution. Lyophilized nanoparticles were stored at −80°C in a sealed container until used.

### Mice Immunization

**Figure 1:**
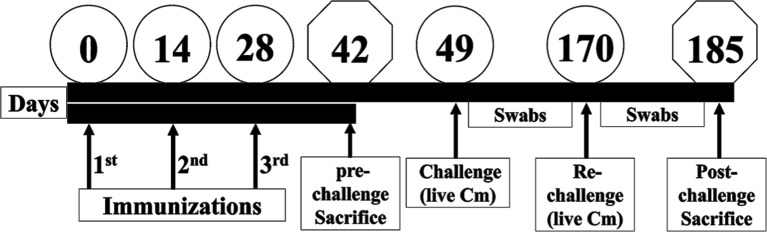
Schematic of mice immunization and challenge studies. Three groups of female BALB/c mice were immunized three times (Days 0, 14 and 28) with either PBS or with 50 µg of PLGA-rMOMP *via* the SC or IN routes. Mice from each group were sacrificed on day 42 (pre-challenge sacrifice). The remaining mice received vaginal challenge with live *C. muridarum* on day 49 (challenge) and day 170 (re-challenge). Cervico-vaginal swabs were collected at 3-day intervals, and mice were sacrificed on day 185 (post-challenge sacrifice).

Female BALB/c mice (4-6 weeks-old) were purchased from Charles River Laboratory (Raleigh, NC) and housed under standard pathogen-free and controlled environmental conditions, and provided with food and water *ad libitum*. Mice were acclimatized for two-weeks before all experimental procedures as approved by Alabama State University Institutional Animal Care and Use Committee (IACUC). Mice used here were a subgroup of our recently published study (13). Mice were divided into experimental groups (10 to 11 mice/group) for the immunization studies and were immunized, as shown in **Figure 1**. Briefly, two groups of mice received either three SC or IN immunizations (pre) at two-week intervals with PLGA-rMOMP. Each mouse received 50 µg/100 µL SC or 50 µg/20 µL IN (10 µL each nostril) of PLGA-rMOMP in sterile phosphate-buffered saline (PBS). Mice in the PBS group were administered SC with 100 µL of sterile PBS. Two-weeks following the last immunization (day 42), 5 mice/group were sacrificed to collect spleen and serum samples for cellular and humoral immune-effectors analyses, respectively.

### *C. muridarum* Vaginal Challenge and Re-Challenge

For the challenge study, SC- or IN-immunized mice (6/group) and PBS (5/group) were each injected SC with 2.5 mg of Depo-Provera two-weeks after the last immunization and one week later (day 49) by an intravaginal challenge with 1×10^5^ IFU of *C. muridarum* in sucrose-phosphate-glutamic acid (SPG) buffer (10). For the re-challenge (post) study, the same mice were injected SC with 2.5 mg of Depo-Provera on day 163 and one week later (day 170) by an intravaginal re-challenge with 1×10^5^ IFU of *C. muridarum*. All mice were sacrificed two-weeks following the re-challenge to collect spleen and serum samples for cellular and humoral analyses, respectively.

### *C. muridarum* Quantification From Vaginal Swabs

Vaginal swabs were collected from each mouse at three-day intervals up to three-weeks after the first *C. muridarum* challenge (day 49) and two-weeks after the re-challenge (day 170). All swabs were collected in SPG buffer and stored at -80°C to quantify *C. muridarum* vaginal load (10). Briefly, McCoy cells were propagated in DMEM) and seeded at (5×10^4^/well) in 96-well cell culture plates. Confluent cell monolayers were inoculated with vaginal swab suspensions containing 0.5 µg/mL cycloheximide, centrifuged at 750g for 1 h at RT, and then incubated for 2 h at 37°C in a 5% CO_2_ humidified atmosphere. After that, fresh media containing 0.5 µg/mL cycloheximide was added to cells and incubated for 30 h. Cells were washed, fixed in 95% ethanol, and stained with a FITC-labeled *Chlamydia* antibody using the Remel™ PathoDx™ *Chlamydia* Culture confirmation kit. All swabs were cultured in duplicates, and 25 fields from each well were captured using an automated Nikon confocal microscope (Melville, NY).

### Antigen-Specific T-Cell Stimulation

Spleens were collected from immunized (day 42) and re-challenged mice (day 185) for T-cell stimulation as described (10, 15). Briefly, spleens were pooled per group and kept in RPMI-1640 supplemented with 10% FBS and antibiotics-antimycotic. Single-cell suspensions were obtained and filtered through a 40-µm nylon mesh strainer and washed before red blood cell lysis using ACK lysing solution. The cells were incubated with anti-CD 90.2-conjugated magnetic beads, and total purified T-cells were isolated by positive selection over MACS columns. Naïve single spleen cell suspensions were treated with mitomycin-C (25 µg/mL) for 30 min at 37°C in a 5% CO_2_ humidified atmosphere and used as antigen-presenting cells (APCs). Purified T-cells (1×10^6^) and APCs (1×10^6^) co-cultures were stimulated with rMOMP (5 μg/mL) in round bottom-polypropylene tissue culture tubes and incubated for 48 h at 37°C in a 5% CO_2_ humidified atmosphere. Cell-free culture supernatants were collected by centrifugation and stored at -80°C for cytokines quantification.

### Cytokines Quantification

Cytokines (IFN-γ, IL-2, IL-10 and IL-17) were quantified in cell-free culture supernatants as described (7, 10). All samples were run in triplicates, and experiments were repeated at least three times.

### T-Cell Proliferation, Memory, and Effector Phenotypes

Purified T-cells were subjected to CFSE-based proliferation assay, as previously reported (7, 10). Briefly, purified T-cells from immunized and re-challenged mice were labeled with CFSE (5 µM) by incubating for 20 min at 37°C in a 5% CO_2_ humidified atmosphere. CFSE labeled T-cells (0.5×10^6^) were co-cultured with APCs (0.5×10^6^) and stimulated with rMOMP (5 μg/mL) in round-bottom polypropylene tissue culture tubes and incubated for 120 h at 37°C. After incubation, the cells were harvested and stained using CD3-APC-Cy7, CD4-PerCP-Cy5.5, CD62L-APC, and CD44-PE to evaluate T-cell proliferation and memory (CD44^high^ CD62L^high^) and effector (CD44^high^ CD62L^low^) phenotypes. Stained cells were washed, fixed, and data were acquired on a BD LSR II flow cytometer and analyzed using FCS Express 6 FLOW (De Novo Software, Pasadena, CA). Gating on CFSE^+^ T-cells was used for the selection of CD3^+^CD4^+^ T-cell populations. Histogram fluorescence intensities were used to quantify the proliferating and resting T-cells amongst the total CFSE^+^CD3^+^CD4^+^ T-cells.

### Serum and Mucosal Antibody Isotypes

Pooled sera or vaginal wash samples from each group of mice were used to quantify rMOMP-specific antibodies IgG isotypes (IgG2a and IgG2b (Th1) and IgG1 (Th2)) and mucosal IgA as described (10, 12, 14, 15). Briefly, ELISA plates were coated with 100 µL (1 µg/mL) of purified rMOMP and kept overnight at 4°C. Plates were washed with PBS-Tween 20 (PBST) and blocked in 3% non-fat dry milk. Samples were serially diluted two-fold, starting at 1:4000 (serum IgG1), 1:500 (serum IgG2a and IgG2b), 1:25 (mucosal wash IgG1, IgG2a, and IgG2b), and 1:5 (mucosal wash IgA) to determine the endpoint titers. Antigen-specific IgG2a and IgG2b (Th1) and IgG1 (Th2) were detected using isotype-specific HRP-conjugated goat anti-mouse antibodies and TMB substrate. The endpoint titer was considered the last sample dilution with readings higher than the mean +5 standard deviations of the negative control serum or vaginal wash (IgG isotypes) or the mean +3 standard deviations of the negative control vaginal wash samples (IgA). All samples were run in triplicates and experiments were repeated at least three times. The ratios for Th1 and Th2 antibodies were calculated from the endpoint titers (**Table 1**) using the following equation:

$$Ratio=\frac{Th1\;(IgG2a\;or\;IgG2b)}{Th2\;(IgG1)}$$

**Table 1:** rMOMP-specific serum antibodies endpoint titers of immunized mice.

Antibodies	PBS****		SC		IN	
	Pre-challenge	Post-challenge	Pre-challenge	Post-challenge	Pre-challenge	Post-challenge
**IgG2a**	-	1,000	128,000	64,000	-	16,000
**IgG2b**	-	-	256,000	128,000	2,000	8,000
**IgG1**	-	-	512,000	128,000	32,000	4,000

‘-’not detected.

### Serum Antibody Isotypes Avidity

Serum antibody isotypes avidity index (AI) was determined as previously described (10, 13). ELISA plates were coated with purified rMOMP, as described above in the serum and mucosal antibodies section. Sera were diluted (1:50, 1:100, 1:200 and 1:400) and then added to wells in parallel (2 sets per plate) and incubated for 2 h at RT. Plates were washed with PBST, and one set for each serum sample was treated with urea (8M in PBST), and the other set was treated with PBST for 5 min at RT. After washing, rMOMP-specific IgG2a and IgG2b (Th1) and IgG1 (Th2) were detected using isotype-specific HRP-conjugated goat anti-mouse antibodies and TMB substrate. All samples were run in triplicates, and experiments were repeated at least three times. The AI was calculated using the following equation:

$$AI(\%)=\left(\frac{ODwithurea}{ODwithouturea}\right)100$$

### Neutralization of *Chlamydia In Vitro*

*C. muridarum* neutralization by serum from immunized and re-challenged mice was performed using McCoy cells as previously described (10). Briefly, McCoy cells were propagated in DMEM and seeded (5×10^4^/well) in 96-well flat-bottom cell culture plates. In a separate 96-well plate, *C. muridarum* elementary bodies (EBs) at 500 IFU/well were treated with optimally diluted serum (1:100) and incubated for 30 min at 37°C on a slowly rocking platform. Treated-EBs were then added to McCoy cells confluent monolayers and centrifuged at 750g for 1 h, followed by incubation for 2 h at 37°C in a 5% CO_2_ humidified atmosphere. The medium was replaced with fresh DMEM containing 10% FBS and 0.5 µg/mL cycloheximide and then incubated for 30 h. Untreated-EBs and naïve serum were, respectively, used as positive and negative controls. After incubation, the plates were fixed with 95% ethanol and stained using the Remel™ PathoDx™ *Chlamydia* culture confirmation kit. All samples were cultured in triplicates, and three fields from each well were captured using an automated Nikon confocal microscope. Experiments were repeated at least two times.

### Statistical Analysis

Data were analyzed by two-way analysis of variance (ANOVA) followed by Tukey’s multiple comparison test to compare recovered IFU, cytokines production, and *in vitro* neutralization between and within groups. One-way ANOVA followed by Holm-Sidak was used for mean IFU comparison between groups, and all analyses were performed using GraphPad Prism 8 (San Diego, CA). *P* values ≤ 0.05 were considered statistically significant.

## Results

### Immunization With PLGA-rMOMP Protects Mice Against Genital *Chlamydia*

**Figure 2:**
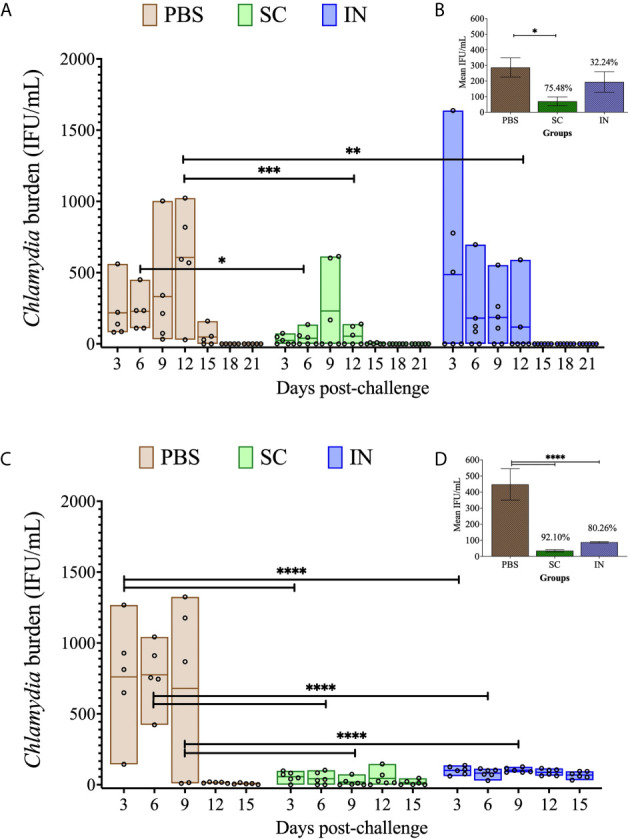
Protection of PLGA-rMOMP-immunized mice against vaginal *C. muridarum* challenge and re-challenge. Groups of mice were each immunized three times at two-week intervals with PLGA-rMOMP *via* the SC or IN routes. Each mouse was challenged intravaginally with 1×10^5^ IFU of *C. muridarum*, and cervico-vaginal swabs were collected and propagated in McCoy fibroblasts to quantify the recovered IFU. Each floating bar represents the minimum and maximum range for IFU counts (IFU/mL) from individual swabs. The middle line represents the mean (IFU/mL) for each group of mice after challenge **(A)** and re-challenge **(C)**. Graph inserts represent IFU/mL (mean ± SE) calculated for combined days 3 to 15 after challenge **(B)** and re-challenge **(D**); numbers above bars indicate percent (%) reduction in recovered IFU compared to the PBS control. Statistical analyses were performed using two-way ANOVA followed by Tukey’s multiple comparisons for IFU/mL **(A, C)** and one-way ANOVA followed by Holm-Sidak for the mean IFU/mL **(B, D).** Significant differences were considered at ****P < 0.0001, ***P < 0.001, **P < 0.01 and *P < 0.05. No exclusions were applied for IFU counts.

We recently reported that PLGA-rMOMP induced heightened adaptive immune responses in mice immunized *via* the SC rather than the IN route (13). Here we investigated the capacity of the PLGA-rMOMP nanovaccine to confer protective efficacy in mice against a *C. muridarum* genital challenge and re-challenge. We tested the hypothesis that the SC rather than the IN-immunization route would be more efficacious in protecting mice against genital *Chlamydia.* Following three immunizations with PLGA-rMOMP *via* the SC or IN routes, mice received an intravaginal challenge with *C. muridarum* (1×10^5^ IFU/mouse) on day 49 to evaluate protection by quantifying chlamydial IFU from vaginal swabs. SC and IN immunization significantly protected (*P* < 0.05 to 0.001) mice against a challenge as compared to the PBS control mice that consistently had higher recovered IFU (**Figure 2A**). Overall, all mice immunized *via* the SC route had lower IFU than the IN group even though the differences did not reach statistical significance (**Figure 2B**). Further, we evaluated whether the nanovaccine can elicit protection against a re-challenge and administered mice an intravaginal *C. muridarum* (1×10^5^ IFU/mouse) 120 days after the first challenge. Both SC and IN immunization significantly (*P* < 0.0001) enhanced protection after re-challenge (**Figures 2C, D**) versus the first challenge (**Figures 2A, B**) by the lower recovered IFU, suggesting elicitation of protection facilitated by the nanovaccine. The recovered IFU from the PBS control mice markedly reduced on day 12 compared to day 15 for the SC- and IN-immunized mice (**Figure 2C**), suggesting possibly an infection-induced immunity-boosting effect arising from the first challenge.

### Induction of Antigen-Specific Cytokines From T-Cells After Immunization and Re-Challenge

**Figure 3:**
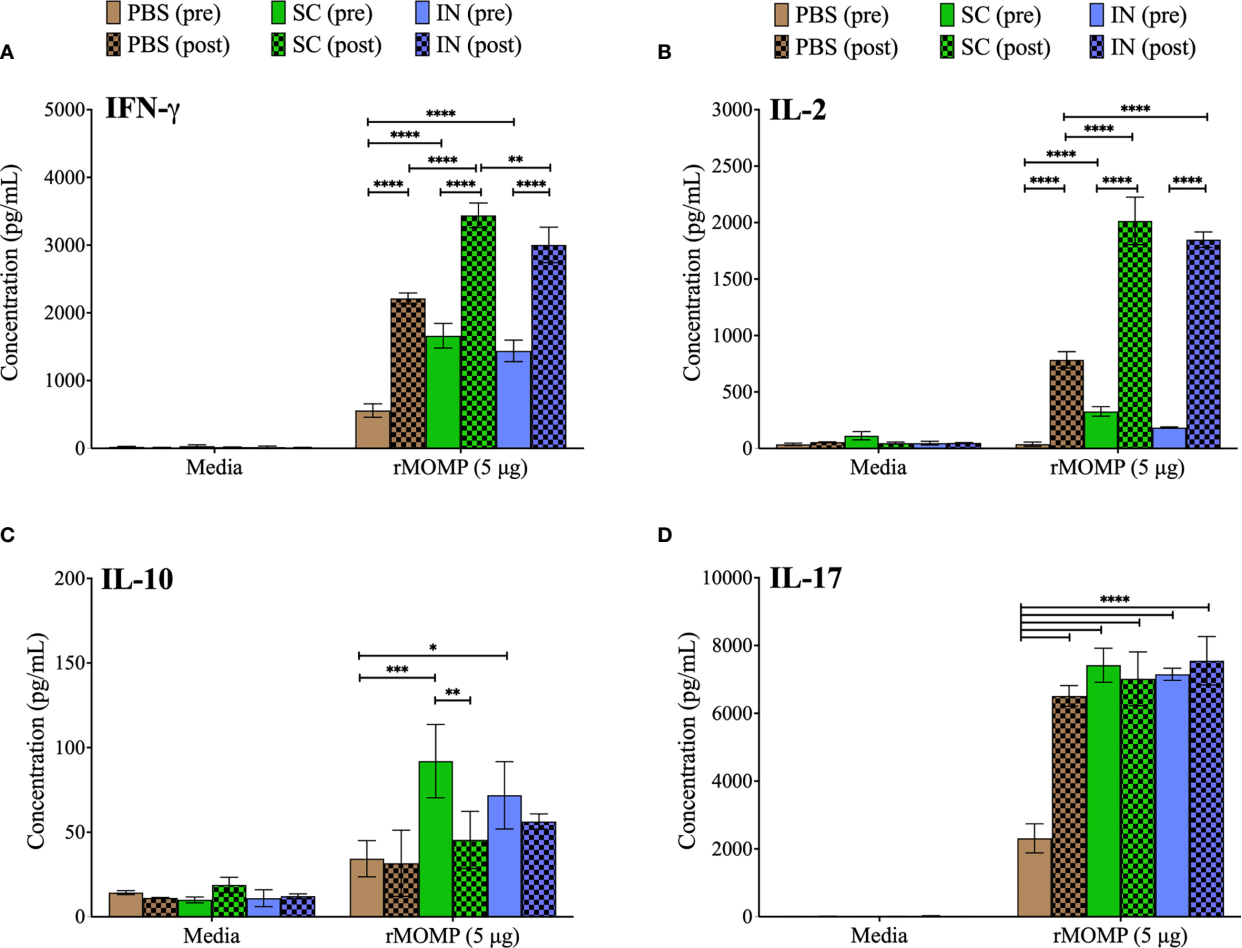
Enhanced production of *Chlamydia-*specific Th1, Th2, and Th17 cytokines by T-cells from PLGA-rMOMP-immunized and challenged mice. Immunomagnetic purified splenic T-cells (1×10^6^) were co-cultured with mitomycin-C treated APCs (1×10^6^) and stimulated with rMOMP (5 μg/mL) for 48 h at 37°C in a 5% CO_2_ humidified atmosphere. Cell-free supernatants were collected by centrifugation and used for quantification of cytokines; IFN-γ (Th1) **(A)**, IL-2 (Th1) **(B)**, IL-10 (Th2) **(C),** and IL-17 (Th17) **(D)** using cytokine specific ELISAs. Immunized mice (pre); immunized-challenged and re-challenged mice (post). Each bar represents the mean ± standard deviation (SD) of triplicates from each sample. Statistical analyses were performed using two-way ANOVA followed by Tukey’s multiple comparisons and significant differences were considered at ****P < 0.0001, ***P < 0.001, **P < 0.01 and *P < 0.05.

Induction of cell-mediated immunity is pivotal in protection against genital *Chlamydia* (16). Thus, we assessed T-cell cytokines that may correlate with the protection of mice against genital *C. muridarum*. We quantified Th1 (IFN-γ, IL-2), Th2 (IL-10) and Th17 (IL-17) cytokines secreted by purified T-cells from mice after PLGA-rMOMP immunization (pre) and again after challenge and re-challenge (post). As illustrated in **Figure 3A**, SC and IN immunized mice (pre) produced ~ 3-fold significantly higher IFN-γ (*P* < 0.0001) compared to the PBS controls. Similarly, IFN-γ production was higher (*P* < 0.0001) in the SC and IN mice (post) relative to the PBS controls. IL-2 was significantly (*P* < 0.001) produced by T-cells from immunized (SC and IN) mice (pre) and by more than a 2-fold increment (*P* < 0.0001) after re-challenge (post) compared to the PBS mice (**Figure 3B**). Though IFN-γ and IL-2 secretions were less in the PBS controls (pre and post), they were increased (*P* < 0.0001) after re-challenge (post) (**Figures 3A, B**). Overall, all mice produced less IL-10 (pre or post); however, IL-10 was reduced (*P* < 0.01) only after re-challenge of the SC mice (**Figure 3C**), therefore confirming a predominantly Th1 response. The results also showed that IL-17, an immunoregulatory cytokine, which is essential in intracellular pathogen clearance (17) was enhanced (*P* < 0.0001) in the SC and IN mice (pre) in comparison to the PBS control mice, and all groups of mice after re-challenge (post) as seen in **Figure 3D**.

### Induction of Antigen-Specific CD4^+^ T-Cell Proliferation and Memory and Effector Phenotypes After Immunization

CD4^+^ T-cell-derived memory and effector phenotypes play important roles in vaccine-induced immunity against *Chlamydia*, as we previously reported (10). We hypothesized that PLGA-rMOMP would promote specific T-cell activation to protect immunized mice against genital *Chlamydia*. T-cells were collected from mice after immunization (pre) and CFSE-labeled. Labeled T-cells were co-cultured with APCs followed by stimulation with rMOMP to evaluate T-cell proliferation along with memory and effector phenotypes employing multi-parameter flow cytometry.

**Figure 4:**
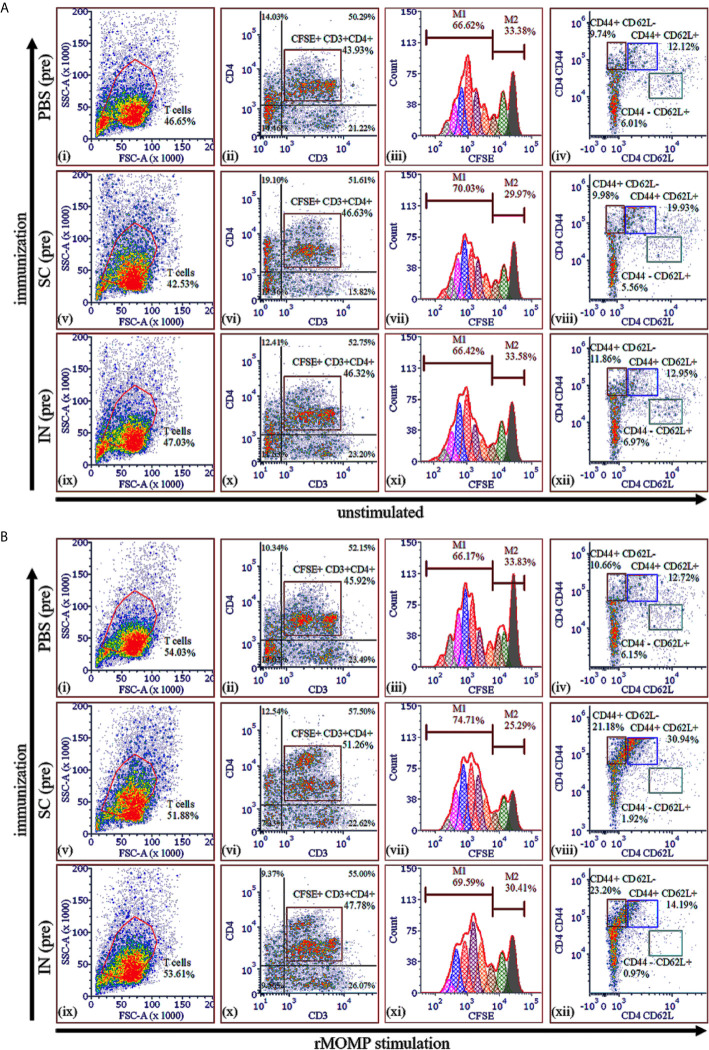
*Chlamydia*-specific T-cell proliferation and memory and effector T-cells in PLGA-rMOMP immunized and challenged mice. Purified T-cells (1×10^6^) labeled with fluorescent CFSE dye were co-cultured with mitomycin-C treated APCs (1×10^6^) and stimulated with rMOMP (5 μg/mL) for 120 h at 37°C in a 5% CO_2_ humidified atmosphere. Co-cultures were centrifuged, and cells were stained with fluorochrome-labeled specific antibodies for CD3, CD4, CD44 and CD62L surface markers. Cells were acquired on a flow cytometer and analyzed by gating on CD3^+^ T-cells with secondary gating on CFSE^+^CD3^+^CD4^+^ T-cells for proliferating memory (CD44^+^ CD62L^+^) and effector (CD44^+^ CD62L^-^) T-cell phenotypes. Immunized mice (pre), unstimulated **(A)** and, rMOMP stimulation **(B)**. Proliferating T-cells (M1) and non-proliferating T-cells (M2).

As illustrated in **Figure 4A**, unstimulated CSFE^+^CD3^+^CD4^+^ T-cells were high in the SC (46.63% (vi)) and IN (46.32% (x)) immunized mice (pre) as compared to the PBS control (43.93% (ii)). Moreover, there were more proliferating CD4^+^ T-cells (M1 population) than resting CD4^+^ T-cells (M2 population) with the SC mice exhibiting higher proliferating CD4^+^ T-cells (70.03% (vi)) as compared to the IN (66.42% (xi)) and PBS control (66.62% (iii)). Following stimulation of cells with rMOMP an increase of CFSE^+^CD3^+^CD4^+^ T-cells was seen more in the SC (51.26%) than the IN (47.78%) and PBS (45.92%) groups, respectively (**Figures 4B** (ii), (vi), (x)). Importantly, we observed an enhancement in the M1 proliferating CD4^+^ T-cells in the immunized SC (74.71%) and IN (69.59%) mice but not the PBS control (66.17%) (**Figures 4B** (iii), (vii), (xi)), which are suggestive of immune memory for antigen recognition. These results suggest increased in T-cell numbers and enhanced proliferation of CD4^+^ T-cells, especially in the SC-immunized mice.

We also quantified memory (CD44^high^ CD62L^high^) and effector (CD44^high^ CD62L^low^) phenotypes in unstimulated and rMOMP-stimulated cells by gating on the CFSE^+^CD3^+^CD4^+^ T-cell populations. Unstimulated proliferating CD3^+^CD4^+^ T-cells from the SC-immunized mice (pre) differentiated into 19.93% memory phenotype (CD44^+^ CD62L^+^) as compared with 12.95% and 12.12%, respectively, for the IN and PBS groups (pre) (**Figures 4A** (iv), (viii), (xii)), suggesting higher induction of memory cells *via* the SC route. Notably, was the marked enhancement of CD3^+^CD4^+^ memory (CD44^+^ CD62L^+^) in SC-mice (30.94%) (**Figure 4B** (viii)) compared to the IN (14.19%) and PBS (12.72%) mice (**Figures 4B** (xii), (iv)). After rMOMP stimulation, the effector T-cell phenotype (CD44^+^ CD62L^-^) increased in both the SC (21.18%) and IN (23.20%), compared to the PBS (10.66%) mice (**Figures 4B** (viii), (xii), (iv)), which may be attributed to immunological memory induced by the nanovaccine. Collectively, the SC rather than the IN route elicited higher cellular-immune responses, thereby correlating with their higher protection against genital *Chlamydia*.

### Induction of Antigen-Specific Systemic and Mucosal Antibody Immune Responses After Immunization and Re-Challenge

**Figure 5:**
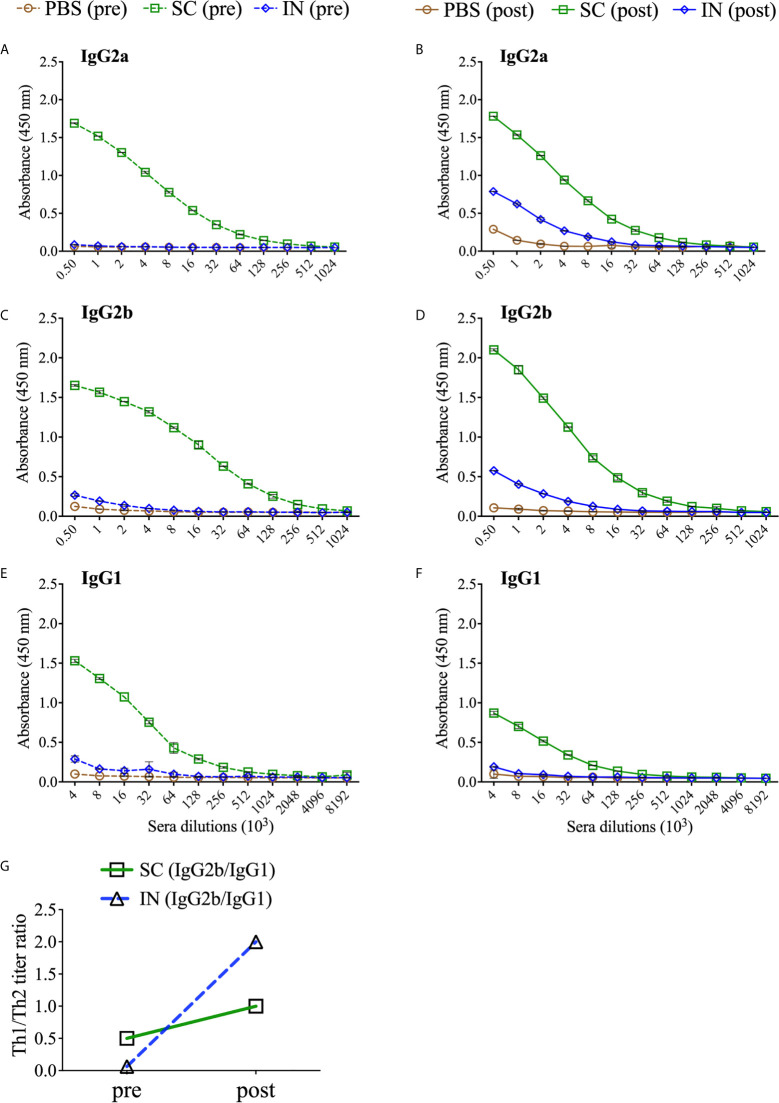
PLGA-rMOMP immunization *via* the SC route induced enhanced antigen-specific serum antibody isotypes. Groups of mice were immunized three-times at two-week intervals with either PBS or PLGA-rMOMP *via* the SC or IN routes. Each mouse was then challenged intravaginally with 1×10^5^ IFU of *C. muridarum*, and pooled sera per group were used to quantify rMOMP-specific antibody isotypes by ELISAs. Immunized mice (pre) sacrificed groups; IgG2a **(A)**, IgG2b **(C)**, IgG1 **(E)**, and immunized-challenge and re-challenge (post) groups; IgG2a **(B)**, IgG2b **(D)**, IgG1 **(F)**. Sera were diluted at a two-fold serial dilution to determine the endpoint antibody isotype titers. Pre and post Th1/Th2 ratio **(G)** for SC and IN groups. Each data point represents the mean ± SD of triplicates from each sample.

The humoral arm of adaptive immunity also plays a significant role in protective immunity against *Chlamydia* (1). The protection afforded by PLGA-rMOMP in immunized mice after the first challenge and re-challenge led us to investigate antigen-specific systemic and mucosal antibodies that may correlate with protection. Sera collected after immunization (pre) and re-challenge (post) of mice were used to quantify rMOMP-specific antibody isotypes by ELISA. In general, SC immunization induced enhanced antigen-specific Th1 (IgG2a and IgG2b) and Th2 (IgG1) antibodies and their specific titers before (pre) and after re-challenge (post) as compared with IN immunization (**Figures 5A–F** and **Table 1**). Re-challenge (post) of the SC mice diminished Th1 and Th2 antibodies compared to those after immunization (pre). Conversely, the IN mice produced enhanced Th1 and not Th2 antibodies (**Figures 5B, D, F** and **Table 1**). The PBS control (pre) mice did not produce any antigen-specific antibodies (**Figures 5A, C, E** and **Table 1**).

Based on the enhanced antibody responses, especially in the SC mice, we next examined the IgG2b/IgG1 Th1/Th2 antibody ratios to discern the impact of the immunization routes on humoral protective immunity. As depicted in **Figure 5G**, the SC, and especially the IN immunization routes triggered a predominant Th2 response (pre). Interestingly, although SC mice produced higher Th1 and Th2 antibodies, a bias towards a predominant Th1 response (post) occurred more in the IN mice by the higher Th1/Th2 ratio. The IgG2a/IgG1 Th1/Th2 ratios were not calculated because of no IgG2a endpoint titer in the IN mice (pre). Overall, these results reveal insights into possible Th1 and Th2 antibody-protective immune effectors elicited by PLGA-rMOMP with a predominant Th2 response (pre) after immunization and then mixed Th1/Th2 (SC mice) and Th1 (IN mice) responses after a chlamydial genital re-challenge (post).

**Figure 6:**
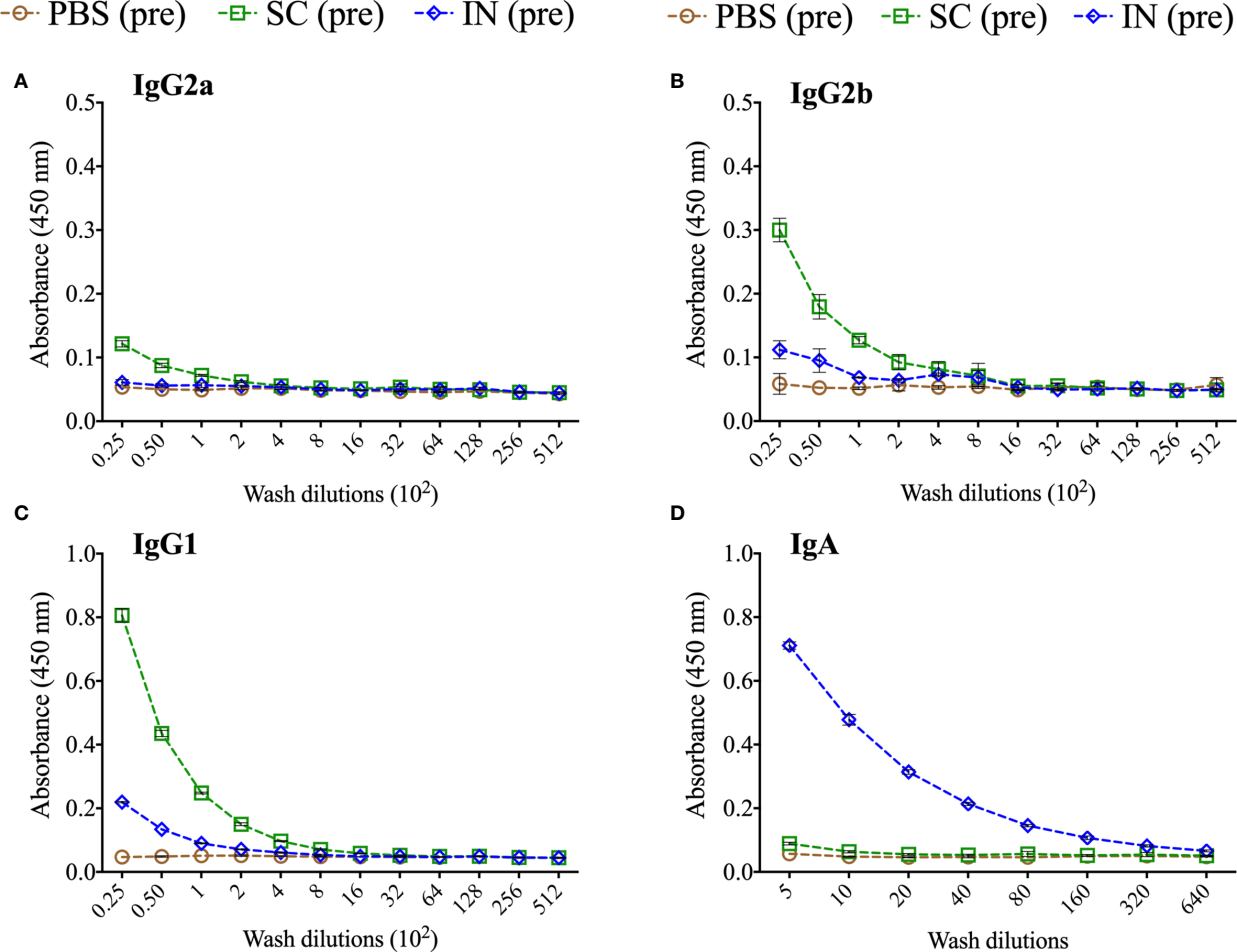
Production of antigen-specific mucosal antibodies in immunized mice. Groups of mice were immunized three times at two-week intervals with either PBS or PLGA-rMOMP *via* the SC or IN routes. Pooled mucosal washes per group were diluted at a two-fold serial dilution to determine by ELISA the endpoint rMOMP-specific antibody isotype titers; IgG2a **(A)**, IgG2b **(B)**, IgG1 **(C)**, IgA **(D)**. Each data point represents the mean ± SD of triplicates from each sample.

We also quantified rMOMP-specific mucosal antibodies in vaginal washes collected from immunized mice (pre). SC immunization induced higher Th2 (IgG1) and lower Th1 (IgG2a and IgG2b) antibody titers, whereas IN immunization only induced a minimal Th2 (IgG1) antibody response (**Figures 6A–C** and **Table 2**). Of no surprise, IN immunization elicited a higher mucosal IgA antibody response and titer than the SC immunization (**Figure 6D**, **Table 2**). The PBS control mice did not produce mucosal antibodies (**Figures 6A–D**. These results indicate that the SC delivery route elicited higher antigen-specific mucosal antibodies (Th1 and Th2), contrastingly to the predominant IgA antibody produced *via* the IN route.

**Table 2:** Antigen specific mucosal-antibodies endpoint titers of immunized mice.

Antibodies	PBS	SC	IN
**IgG2a**	–	25	–
**IgG2b**	–	100	–
**IgG1**	–	200	50
**IgA**	–	5	320

‘-’not detected.

### Avidity-Index (AI) of Antigen-Specific Serum Antibody Isotypes After Immunization and Re-Challenge

**Figure 7:**
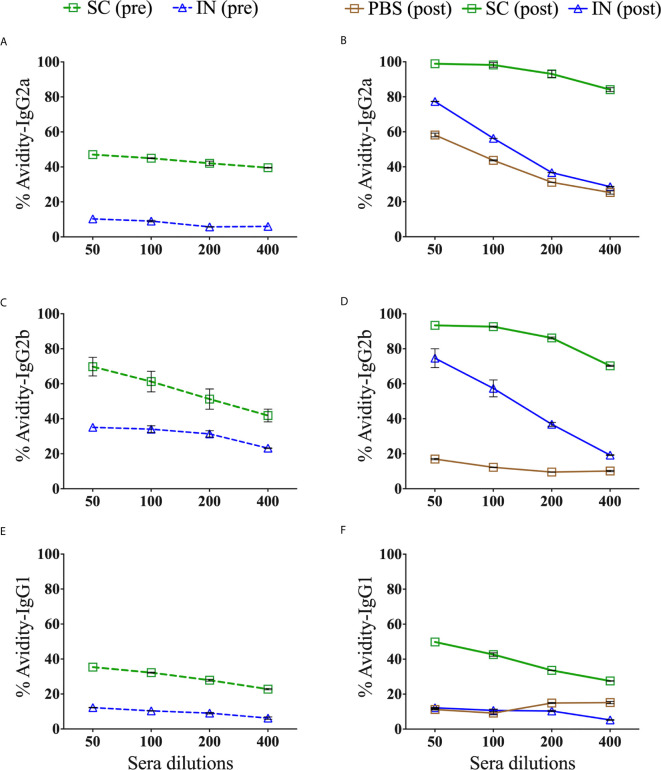
Avidity index of antigen-specific serum IgG2a, IgG2b and IgG1 isotype antibodies. Groups of mice were immunized three times at two-week intervals with PBS or PLGA-rMOMP *via* the SC or IN routes. Each mouse was challenge and re-challenge intravaginally with 1×10^5^ IFU of *C. muridarum.* Avidity-ELISAs were conducted using pooled sera from immunized (pre) and immunized-re-challenge (post) mice to determine the avidity index (%) for rMOMP-specific IgG2a **(A)**, IgG2b **(C)**, IgG1 **(E)** antibodies (pre), and IgG2a **(B)**, IgG2b **(D)**, IgG1 **(F)** antibodies (post). Each data point represents the mean ± SD of triplicates from each sample. The PBS control mice (pre) did not produce antigen-specific antibodies to calculate the avidity index **(A, C, E)**.

The binding strength of a specific antibody to a selective epitope enhances the functionality of antibody immune responses. Chlamydial MOMP contains antigenic epitopes that are important in inducing specific antibodies (18). To determine the isotype differences in functional Th1 (IgG2a and IgG2b) and Th2 (IgG1) antibodies induced by immunization (pre) and re-challenge (post), we used urea as a chaotropic agent for the elution of low-avidity serum antibodies and calculated the AI (%). Our results demonstrate that the SC mice (pre and post) produced more highly functional avidity antibodies than the IN mice (**Figure 7**). The functional avidity antibodies were in the order of magnitude IgG2b (**Figure 7C**), IgG2a (**Figure 7A**), and then IgG1 (**Figure 7E**). Following a re-challenge (post) of the SC mice, the functional IgG2b and IgG2a (**Figures 7D, B**) avidity antibodies improved compared to a slight increase in IgG1 (**Figure 7F**). On the other hand, the IN-immunized mice (pre) selectively produced functional IgG2b avidity antibodies, which improved after re-challenge (post) (**Figure 7D**). Interestingly, a re-challenge of the IN mice (post) also heightened the functionality of IgG2a (**Figure 7B**) but not of IgG1 (**Figure 7F**) avidity antibodies. Re-challenging the PBS control (post) triggered only functional IgG2a avidity antibodies, albeit lower than that induced by the SC and IN mice (**Figure 7B**). The PBS control mice (pre) did not produce antigen-specific antibodies (endpoint titers in **Table 1**) to calculate the avidity index (**Figures 7A, C, E**). Overall, the data shows that the production of antigen-specific functional avidity antibodies is dependent on the delivery route, given the heightened functional Th1 than Th2 avidity antibodies induced in SC mice.

### Immunization and Re-Challenge of Mice Induced Serum Antibodies That Neutralized *Chlamydia* Cellular Infectivity *In Vitro*

**Figure 8:**
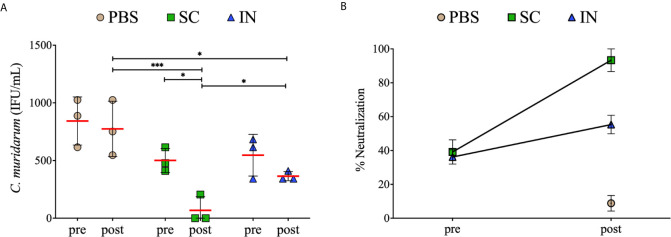
Serum-mediated neutralization of *Chlamydia* infectivity of mouse fibroblasts. McCoy fibroblasts were infected with sera-*C. muridarum* inoculum in triplicates in 96-well plates and incubated for 30 h. Cells were fixed and stained, and IFU images were captured by immunofluorescence microscopy. Results are shown as *C. muridarum* (IFU/mL) detected in cells exposed to pooled sera from groups of mice. Each symbol represents the IFU counts (mean ± SD) of three fields and three different wells from immunized (pre) and immunized re-challenge (post) groups of mice. Horizontal red line represents the mean IFU/mL per group **(A)**. The % neutralization (mean ± SE) of *C. muridarum* EBs in fibroblasts in the presence of serum antibodies **(B)**. Statistical analyses were performed using two-way ANOVA followed by Tukey’s multiple comparisons and significant differences were considered at ***P < 0.001and *P < 0.05.

Neutralizing antibodies play an essential role in *Chlamydia* infection by binding on the EBs surface. Therefore, we assessed the serum antibody-mediated neutralization of chlamydial EBs *in vitro*, using immune (pre) and re-challenge (post) sera from mice. EBs pre-incubated with sera)* *were added to McCoy fibroblasts and incubated for 30 h to assess infectivity by quantifying the IFU. The results in **Figure 8A** demonstrate that the SC- or IN-immunized mice (pre) produced antibodies that neutralized chlamydial EBs infectivity. However, the antibodies produced after re-challenge in the SC mice (post) were more potent in neutralizing EBs (*P* < 0.001) than those of the IN and PBS groups (post). Of significance, re-challenge (post) significantly (*P* < 0.05) enhanced the serum-neutralizing antibodies in the SC but not the IN mice.

Further analysis showed that sera from the SC- or IN-immunized mice (pre) neutralized EBs by 40% and 35%, respectively, relative to the PBS mice (pre) (**Figure 8B**). Moreover, sera obtained from the SC mice after re-challenge (post) markedly neutralized EBs by 91% compared to 56% for the IN mice and 8% for the PBS control (**Figure 8B**). Overall, the results demonstrate that SC and IN immunization, especially the SC, stimulated superior neutralizing antibodies that effectively reduced *Chlamydia* cellular infectivity.

## Discussion

Developing vaccines is a critical process with stringent provisions that the vaccine must stimulate robust immune responses, above those of natural infections, to prevent primary and secondary infections (2, 19). Notwithstanding, there are significant hurdles with subunit vaccines, including premature degradation, efficient delivery system, optimal doses to induce adequate immune responses, and an adjuvant to induce stronger immune responses (20). Given some of the hurdles mentioned above, there has been an increasingly optimistic approach with a different paradigm employing nanoparticles as alternative delivery systems for subunit vaccines. Perhaps this is due to the flexibility of the nanoparticle-based vaccine formulations for enhanced delivery, which may be passive by encapsulating or targeted by adsorbing on the surface of nanoparticles (8). Besides, employing the nanoparticle-delivery system for encapsulating antigens affords stability and protection from premature degradation and the direct intracellular delivery to APCs (20). Accordingly, this approach, employing biodegradable nanoparticles as subunit vaccine delivery systems, has been reported recently for cancer (21, 22) and microbial diseases (4, 20). Since biodegradable nanoparticles undergo degradation by either hydrolysis or enzymatic processes, they can provide a sustained-release of antigens over a prolonged time. Hence, a smaller dose of the released antigen from degrading nanoparticles is potent to stimulate robust immune responses due to delivery efficiency at target sites (8). Along with antigen delivery, nanoparticles provide a concomitant adjuvanting effect (23–27), though the mechanism of adjuvanticity is not well-understood (28, 29).

Amongst the family of biodegradable nanoparticles, PLGA polymers are receiving much scrutiny as delivery platforms for antigens due to their safety, routes of administration, FDA approved, and potentiating properties (12, 13, 30–33). Numerous studies have shown that the PLGA delivery-system can dramatically enhance innate and adaptive immune responses for encapsulated antigens (12, 13, 30, 32, 34–36). Recently, Khademi et al. demonstrated a *Mycobacterium tuberculosis* subunit vaccine formulated with the PLGA: DDA hybrid nanoparticles induced Th1 immune responses (IFN-γ and IgG2a) (37). Similarly, Kabiri and colleagues observed that PLGA delivery of the Human T-cell leukemia virus antigenic epitopes triggered a predominant Th1 (IFN-γ and IgG2a) over Th2 (IL-4 and IgG1) immune responses in mice immunized *via* the mucosal and systemic routes (38). As seen, these studies reinforce the notion that the PLGA delivery system enhances immune responses in favor of encapsulated antigens.

*Chlamydia* infections can lead to reproductive morbidities that arise from severe inflammation, especially at the later stages (39), when antibiotics therapy has reduced efficacy. Notwithstanding, after over 70 years of enduring efforts, an FDA-approved chlamydial vaccine remains an elusive goal (5). Chlamydial recombinant subunit vaccines are currently the focus of vaccine development, with safety and efficiency being foremost, given the exacerbated diseases produced by whole organism-based vaccines (20). The current study proves that immunity elicited by the extended-releasing PLGA-rMOMP nanovaccine can protect against a genital challenge and re-challenge in the mouse model.

Our results demonstrate that irrespective of the PLGA-rMOMP immunization route (SC or IN), mice were significantly protected against a *C. muridarum* intravaginal challenge compared to the PBS controls in terms of their lower IFU. SC immunization afforded better protection after the first *C. muridarum* challenge evidently by the lower IFU in all mice conversely to the higher and varying IFU in the IN-immunized mice. The variability in *Chlamydia* IFU observed between mice on pre-selected days after the *C. muridarum* challenge has been reported (10, 40). However, we did observe an earlier IFU reduction in the PBS controls after re-challenge (day 12), suggesting a possible infection-induced immunity booster effect from the first challenge to afford their early reduced bacterial burden. Enhanced protection against *C. muridarum* re-challenge in the SC- and IN- immunized mice compared to the PBS control is a strong implication of the extended-releasing nanovaccine coordinated with an infection-induced immunity-boosting effect, which facilitated enhanced protection of mice against re-challenge. In conjunction with our observations, other investigators have reported mice protection against a *Chlamydia* re-infection, but for a lesser duration of only 28 days (41) and 92 days (42) between the first and second infections.

Here our results suggest that T-cells are essential mediators of protective immunity ensuing from the immunization of mice with PLGA-rMOMP for adequate protection against a *C. muridarum* challenge and re-challenge. The heightened protective chlamydial-specific Th1 cytokines (IFN-γ and IL-2) and CD4^+^ memory and effector T-cells produced after immunization of mice (SC and IN) corroborate reports of Th1 immune effectors that can clear a *C. muridarum* vaginal challenge (10, 40). Indeed, the protective role of Th1 cytokines has directly been correlated with IFN-γ-mediated *Chlamydia* killing by activating T-cells (43, 44). In line with our observation, CD4^+^ T-cells driven-immune responses with pronounced IFN-γ production are reportedly adequate for protection against *C. muridarum* infections in the genital tract (45–47). More importantly and from a translational perspective, our results are aligned with a study conducted with *C. trachomatis*-infected women that showed a *Chlamydia*-specific CD4^+^ derived IFN-γ response is protective against re-infection and is a crucial adaptive immunity component (48).

Even though IL-2 has no direct effector function, it is directly associated with effector T-cell proliferation, consistent with previous findings of CD4^+^ effector T-cells expansion (10, 49). Studies have shown that the additional IL-2 can boost the antigen-specific T-cell responses, proliferation, and differentiation process (50). The increase in IL-2 production in the current study may be associated with PLGA-rMOMP inducing rMOMP-specific CD4^+^ T-cells proliferation to differentiate into memory (CD44^high^ CD62L^high^) and effector (CD44^high^ CD62L^low^) phenotypes. The expansion of the CD4^+^ T-cell phenotypes indicates that the effective delivery of the nanovaccine within APCs and presentation *via* MHC II (51) may have accounted for enhanced protection, especially in the SC-immunized mice. Confirming this observation, we recently reported the upregulation of *Chlamydia*-specific memory and effector cells mediated by MHC II and CD4^+^ T-cells in mice immunized with PLA-PEG encapsulating the M278 peptide from MOMP or with PLGA-rMOMP (7, 10, 13). Another observation worth mentioning is the upregulated IL-17 in immunized mice, both pre- and post-challenge. This finding is supportive of the immunoregulatory role of IL-17 in host defense against intracellular pathogens (17, 52, 53), and presumably, *Chlamydia*, as shown here. Reportedly, SC immunization of mice with CTH522/CAF01 induced IFN-γ and IL-17, which were associated with protection against chlamydial genital infection and re-infection (42). Others have shown that mucosal immunization of mice with MOMP VS2/4-CTA1-DD reduced chlamydial genital infection and up-regulated IFN-γ and IL-17 producing T cells (54). Moore and colleagues demonstrated that IL-17 promoting Th17 differentiation occurs under the influence of a pro-inflammatory cytokine environment that was reduced in IL-6-deficient DCs (53). Thus, IL-6 is a crucial regulator of Th17 differentiation, which accentuates our previous observations, whereby DCs produced elevated levels of IL-6 when stimulated by rMOMP or its peptide either alone or encapsulated (7, 13).

Although less credence is generally ascribed to the role of antibodies against *Chlamydi*a clearance, many studies suggest that mobilization of both humoral and cellular immune responses are required (47, 55, 56). The abundant expression of MOMP in the biphasic lifecycle of *Chlamydia* is strong evidence for its induction of protective serum neutralizing antibodies (57). Morrison et al. established using B-cell knockout mice that B-cells and CD4^+^ T-cells are necessary for protective immunity in the case of *Chlamydia* re-infection (47). Li et al. demonstrated that B-cells play a distinct role by enhancing *Chlamydia*-specific CD4^+^ T-cells priming within lymph nodes leading to robust production of cytokines (IFN-γ and IL-17) and long-term memory formation (58). Here in this study, concurrent induction of cellular and humoral immune-effectors may have provided the requisites protective immunity against genital *Chlamydia* in mice. Significantly higher production of systemic Th1 (IgG2a and IgG2b) and Th2 (IgG1) *Chlamydia*-specific antibodies and their titers were more notable in the SC rather than the IN delivery of the nanovaccine. Consistent with our results is evidence supporting that PLGA delivery of antigens induces higher production of antibodies *via* the SC- than the IN-immunization route (9, 59). Not surprising was the high production of mucosal IgA only in the IN mice. It could be conjectured that humoral protective immune mechanisms differ in the IN versus the SC mice. For example, the differences could be due to functionally different antibodies like mucosal IgA in the IN and more systemic and mucosal Th1 and Th2 antibodies in the SC mice. Reportedly, high levels of IgG antibodies and T-cells can facilitate early control of chlamydial infections, but the presence of antigen-specific IgA further accelerates the clearance (60). Other investigators also show IgA’s contributory role in protective immunity and efficacy against *Chlamydia* infection in mice (54, 61, 62).

The participation of memory B-cells in humoral protective immunity requires an antigen-specific affinity for re-stimulation to proliferate and differentiate into antibody-producing plasma cells (63). It is well-known that repeated exposure to antigen leads to elevated antibody titers and functionality due to somatic hypermutations and antigen selection in germinal centers where antibody avidity maturation occurs (64). Extended-release of antigens resembles a persistence infection, which drives memory B-cells to re-program for efficient binding and inactivation of the pathogen (65). Serum-neutralization of *Chlamydia* infectivity *in vitro* is employed frequently as a predictor of the vaccination effect (10, 66) since this process is mediated by functional antibodies induced by immunization. Production of functional antibodies leads to memory B-cells formation and correlates directly with a high avidity-index. Our results show that highly functional rMOMP-specific isotype avidity antibody polarization (Th1 or Th2) may have contributed to neutralizing *Chlamydia* infectivity in the SC and IN mice. SC immunization triggered superior Th1 and Th2 functional antibodies but unequivocally more highly functional Th1 (IgG2a and IgG2b) avidity isotypes whose functionality improved after re-challenge. High rMOMP-specific functional avidity antibodies, especially in the SC-immunized mice, may, in part, explain their better protective efficacy. Previously, we showed that the functionality of antibodies becomes stronger with *Chlamydia* challenge in mice immunized SC with a MOMP peptide (M278) encapsulated within PLA-PEG nanoparticles (10).

In conclusion, an advantage of biodegradable nanoparticles is the versatility of the delivery routes for delivering subunit vaccines against mucosal pathogens, as highlighted here with PLGA-rMOMP. Parallel induction of cellular and humoral immune-effectors by the nanovaccine plus an infection-induced immunity-boosting effect orchestrated by immunological memory involving T- and B-cells most likely afforded protective immunity against a *C. muridarum* re-challenge. SC and IN immunization bolstered a dominant Th1 cellular with mixed Th1/Th2 antibody responses, functional Th1 avidity, and neutralizing antibodies. However, the SC route was more efficient in eliciting superior immune-effectors for better protection. PLGA 85:15 delivery of rMOMP protected mice against genital *Chlamydia* facilitated by the extended-release of rMOMP for continuous stimulation of immune cells along with an infection-induced immunity booster after re-challenge. This study is the first to report the protective efficacy conferred by extended-releasing PLGA 85:15 encapsulation of rMOMP against genital *Chlamydia* in the mouse model.

## Data Availability Statement

The original contributions presented in the study are included in the article/supplementary material. Further inquiries can be directed to the corresponding author.

## Ethics Statement

The animal study was reviewed and approved by Alabama State University’s Institutional Animal Care and Use Committee.

## Author Contributions

RS, SD and RV designed and performed experiments. RS analyzed data, wrote and edited manuscript. SD, RV, SAD, LS and GHG performed formal analysis and edited the manuscript. SRS and VAD provided the funding, resources, reviewed and edited the manuscript. VAD as the principal investigator conceptualized and supervised the study.

## Funding

This research was supported by the National Institute of Allergy and Infectious Diseases of the National Institutes of Health under Award Number R21AI111159, NIH-NIGMS-RISE (1R25GM106995-01) and the National Science Foundation (NSF)-CREST (HRD-1241701) and NSF-HBCU-RISE (HRD-1646729) grants. The content of this study is solely the responsibility of the authors and does not necessarily represent the official views of the National Institutes of Health.

## Conflict of Interest

The authors declare that the research was conducted in the absence of any commercial or financial relationships that could be construed as a potential conflict of interest.
